# Diagnostic Accuracy of Electrodiagnostic Comparative Latency Studies of Carpal Tunnel Syndrome: Single Test and Concordance Between Multiple Tests

**DOI:** 10.3390/diagnostics15222888

**Published:** 2025-11-14

**Authors:** Ahmad R. Abuzinadah

**Affiliations:** 1 Department of Neurology, Faculty of Medicine, King Abdulaziz University, Jeddah 21589, Saudi Arabia; aabuzinadah@kau.edu.sa; 2 Neuromuscular Medicine Unit, King Abdulaziz University Hospital, King Abdulaziz University, Jeddah 21589, Saudi Arabia; 3 Department of Internal Medicine, Neurology Division, International Medical Center, Jeddah 23214, Saudi Arabia

**Keywords:** carpal tunnel, diagnosis, electrodiagnostic, predictive value

## Abstract

**Background:** The optimal number of electrodiagnostic tests required to confirm carpal tunnel syndrome (CTS) has not been systematically evaluated. While single comparative latency study (COLS) is commonly used, it remains unclear whether diagnostic accuracy improves when concordance between multiple COLSs is required. **Methods:** We retrospectively reviewed the electrodiagnostic data of patients referred to our center with upper limb symptoms. Diagnostic accuracy was assessed for individual COLSs—median–ulnar mixed palmar latency difference (palmdiff), median–ulnar ring finger latency difference (ringdiff), and median–radial thumb latency difference (thumbdiff)—and for concordance between two COLSs. Sensitivity, specificity, positive predictive value (PPV), and negative predictive value (NPV) were calculated within diabetes mellitus (DM) and non-DM groups. **Results:** We included 538 patients, of whom 305 had CTS and 109 had DM. Among patients without DM, the PPV ranged from 87.6% to 92.6% for single COLS and 94.1% to 94.8% for concordance between two abnormal COLSs. When only patients with symptom durations of >6 months were considered, the PPV of concordance between two abnormal COLSs was consistently 100%. Among patients with DM who were younger than 60 years, the PPV for a single COLS was >89%, and that for concordance between two abnormal COLSs was >94%, whereas in those aged ≥60 years, PPVs dropped to 71%–83% for both strategies, and specificity remained high (>90%) only for concordance between two abnormal COLSs. **Conclusions:** Concordance between two abnormal COLSs enhances diagnostic precision for CTS, particularly in patients without DM and in patients with DM under 60 years of age. In patients with DM aged ≥60 years, the diagnostic accuracy of COLSs was low.

## 1. Introduction

Electrodiagnostic (EDX) testing remains a standard for confirming carpal tunnel syndrome (CTS) in clinical practice as outlined by the American Association of Neuromuscular and Electrodiagnostic Medicine (AANEM)’s practice parameters [1,2]. Conventional EDX parameters include median nerve sensory latency, median motor distal latency, and comparative latency studies (COLSs), such as the median–ulnar mixed palmar latency difference (palmdiff) test, the median–radial thumb latency difference (thumbdiff) test, and the median–ulnar ring finger latency difference (ringdiff) test. COLSs are considered to be more sensitive than an absolute median latency test [3,4]. These tests are sensitive in terms of detecting focal median neuropathy at the wrist, but diagnostic performance varies between parameters, and false results can occur, particularly in populations with polyneuropathy, such as those with diabetes mellitus (DM) [5,6]. EDX parameters, including COLSs, were found to be more sensitive than ultrasounds for diagnosing CTS [7,8]. Additionally, ringdiff was found to have the highest diagnostic accuracy for CTS in patients with diabetic neuropathy when compared to EDX and ultrasound parameters [6].

Several studies have suggested that requiring concordance between multiple EDX tests may enhance diagnostic accuracy [9]. The agreement between two COLSs is adequate for confirming CTS and reduces the rate of false-positive and false-negative results [1,9]. Current guidelines recommend starting with median sensory and motor nerve conduction studies across the wrist. If these results are normal, additional testing with comparative or segmental techniques (or a combination of both) is advised. These approaches have demonstrated high diagnostic performance, with sensitivity typically around 80–90% and specificity exceeding 95% [10,11]. The existing literature has several limitations, however. First, positive predictive value (PPV) and negative predictive value (NPV) for concordance between two COLSs have been assessed using healthy controls rather than using controls from clinic populations. PPV represents the probability that a patient truly has the disease when the test result is positive, whereas NPV reflects the probability that a patient is disease-free when the test result is negative. Since both PPV and NPV are highly dependent on disease prevalence, their accurate determination requires data from broadly representative populations that resemble the clinical practice to minimize bias. PPV is highly dependent on disease prevalence because a fixed false-positive rate produces vastly different absolute numbers in low- versus high-prevalence settings. For example, with a 5% false-positive rate, a low-prevalence disease (1 case per 100,000) would yield nearly 5000 false-positive cases but only one true-positive case, resulting in a very low PPV. In contrast, in a higher-prevalence disease (5000 per 100,000), the same 5% false-positive rate would generate about 4750 false-positive cases but approximately 4500 true-positives, leading to a markedly higher PPV [12]. Second, previous studies did not stratify sensitivity and specificity according to the presence of DM. Third, the diagnostic accuracy of different combinations of COLSs, such as palmdiff with ringdiff or palmdiff with thumbdiff, has not been evaluated.

This study addresses this gap by reporting the diagnostic accuracy of a single COLS (thumbdiff, palmdiff, or ringdiff) and concordance between two COLSs for confirming CTS diagnosis. Our goal is to provide evidence that guides clinical practice as to when a multi-test concordance strategy may be preferable to confirm CTS diagnosis.

## 2. Methods

### 2.1. Study Design and Participants

This was a retrospective study where data were obtained through a chart review of prospectively maintained data from January 2017 to July 2024 at King Abdulaziz University Hospital (KAUH) and the International Medical Center (IMC). The study protocol received approval from the institutional review boards of both centers. All consecutive patients referred to the Clinical Neurophysiology Laboratory (CNPL) for evaluation of upper limb pain, paresthesia, or sensory disturbance were screened for eligibility. All patients were interviewed by the neurologist before conducting EDX using a list of predetermined questions, and the responses were documented in the EDX report.

Participants were included if they met the following inclusion criteria: (1) being 14–80 years of age; (2) having been referred to the CNPL with pain or sensory disturbance in the upper limb; (3) having had CTS considered as a potential differential diagnosis; (4) having available COLS results (palmdiff, thumbdiff, and ringdiff). The exclusion criteria were (1) the presence of weakness in muscles outside the hand and (2) the absence of sensory responses in EDX tests. To maintain the statistical independence of observations, patients were asked specifically in which hand the complaint was more prominent, and only the more symptomatic hand was included for patients with bilateral symptoms.

### 2.2. Definitions of Variables

CTS was diagnosed when at least two of the following three criteria were met: (1) the presence of nocturnal paresthesia; (2) symptom aggravation was experienced during manual tasks such as driving, cycling, or phone use; and (3) symptoms being relieved by hand shaking (positive flick sign) [13,14]. Many investigators support the use of clinical diagnosis to serve as the reference standard for diagnosing CTS [10,15]. We applied these criteria because they have been associated with favorable outcomes following carpal tunnel decompression surgery in randomized clinical trials [13]. On the other hand, sensory symptoms in the median nerve distribution and Phalen’s test were not included, as the available literature indicates that they may result in high false-positive and false-negative rates [11,16,17]. Severity of CTS were described according to Padua and Bland classification [18,19].

Non-CTS cases were defined as those that did not meet the above clinical diagnostic criteria for CTS or that demonstrated signs or symptoms of musculoskeletal conditions, ulnar neuropathy, or cervical radiculopathy. EDX parameters were not used to define CTS and non-CTS cases.

Our study used a non-selective clinic cohort that included all consecutive eligible patients with and without CTS, without predefined control numbers or the recruitment of healthy volunteers from outside the clinic population. Consequently, the non-CTS cases represented the real-world diagnostic challenge of distinguishing CTS from its clinical mimickers. Moreover, this approach reflects the true disease prevalence and captures the full spectrum of disorders that can resemble CTS.

### 2.3. Electrodiagnostic Data Collection

**Figure 1:**
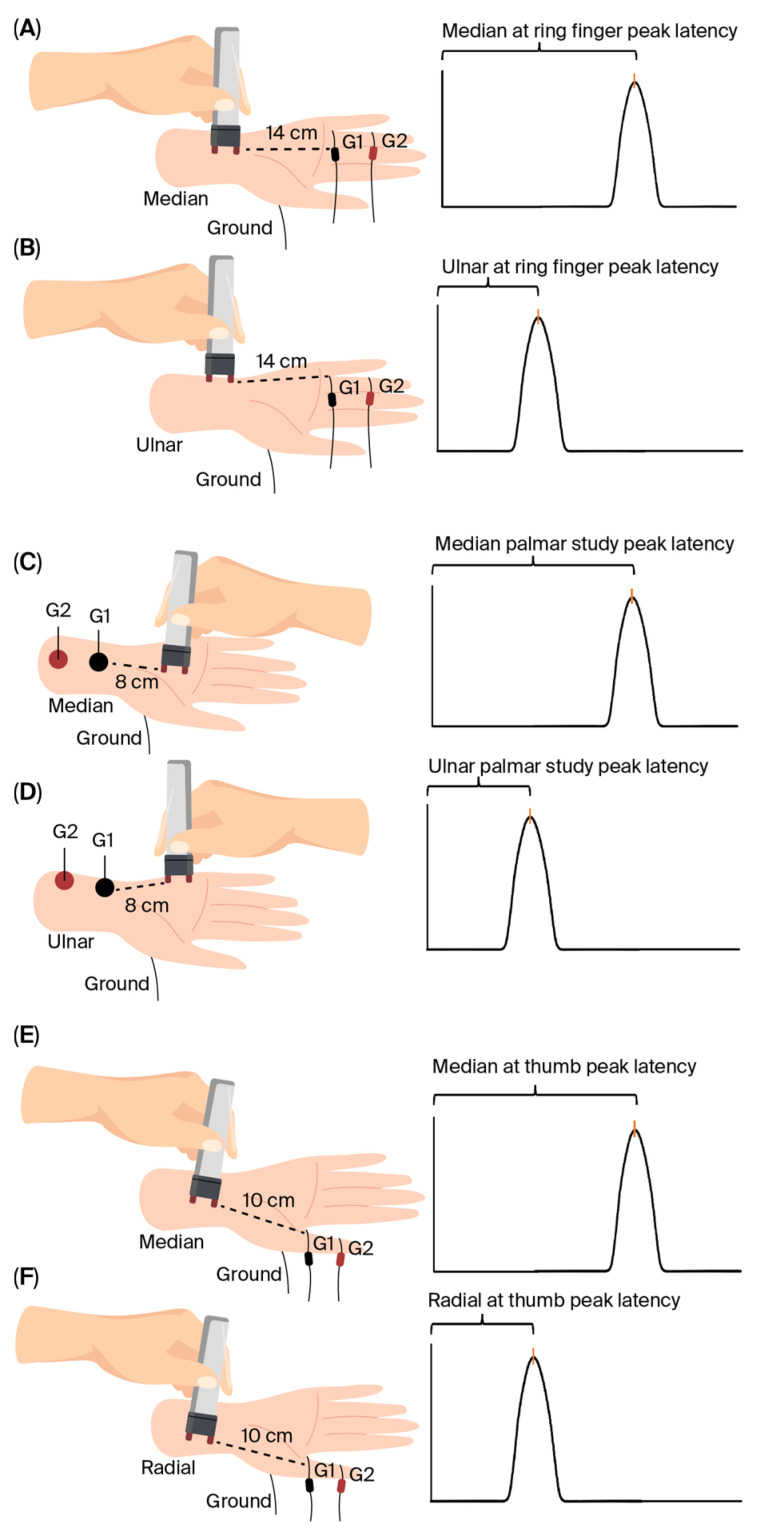
Illustration of the method of obtaining a comparative latency study (COLS). (**A**,**B**): Median–ulnar latency difference determined at the ring finger (ringdiff test). (**C**,**D**): Median–ulnar latency difference determined at the palm (palmdiff test). (**E**,**F**): Median–radial latency difference determined at the thumb (thumbdiff test). G1: Recording electrode. G2: Reference electrode.

Our earlier publication [20] describes in detail the methodology for acquiring the EDX data. Figure 1 illustrates the methods for obtaining COLS. The EDX data were collected as described below.

(A) Median sensory latency measured at digit II (digit II latency).
(B) Comparative latency studies (COLSs) consisting of the following:
  1. Median–ulnar latency difference determined at the ring finger (ringdiff) test (Figure 1A,B);
  2. Median–ulnar latency difference determined at the palmar (palmdiff) test (Figure 1C,D);
  3. Median–radial latency difference determined at the thumb (thumbdiff) test (Figure 1E,F).

Peak latency was used for the sensory studies. All patients who had all three COLSs were included in the diagnostic accuracy analysis whether digit II latency was prolonged or not. The reason for including patients with prolonged digit II latency is that the prolongation of digit II could be part of neuropathy if the selective involvement of the median nerve was not demonstrated through comparative studies. The abnormal cutoffs for COLSs were based on our previous report; see Table 1 [20].

### 2.4. Outcomes

We aimed to report the diagnostic accuracy of the following EDX findings for CTS using the receiver operator characteristic (ROC) area under the curve as well as the sensitivity, specificity, PPV, and NPV.

(A) Single-test strategies were defined by an abnormal result in one parameter:
  1. Palmdiff;
  2. Thumbdiff;
  3. Ringdiff.
(B) Concordance between multiple tests was defined as follows:
  1. One of three: at least one abnormal result among thumbdiff, palmdiff, and ringdiff;
  2. Two of three: at least two abnormal results among thumbdiff, palmdiff, and ringdiff;
  3. One of palm–radial: at least one abnormal result among palmdiff and thumbdiff;
  4. Two of palm–radial: both palmdiff and thumbdiff are abnormal;
  5. One of palm–ring: at least one abnormal result among palmdiff and ringdiff;
  6. Two of palm–ring: both palmdiff and ringdiff are abnormal;
  7. One of radial–ring: at least one abnormal result among thumbdiff and ringdiff;
  8. Two of radial–ring: both thumbdiff and ringdiff are abnormal.

These outcomes were applied separately to the DM and non-DM groups.

### 2.5. Statistical Analysis

Demographic characteristics were summarized using medians, interquartile ranges (IQRs), and frequencies. Comparisons of medians and proportions were performed using the Mann–Whitney U test and the χ^2^ test, respectively. Diagnostic performance for CTS was assessed using ROC curve analysis. Sensitivity, specificity, PPV, and NPV were calculated as measures of diagnostic accuracy. The kappa statistic was used to assess the level of agreement between different comparative latency studies (COLSs), while McNemar’s test was applied to determine whether a significant difference existed between single and concordant COLS in terms of the diagnostic classification of cases. All statistical analyses were conducted with STATA version 13 (StataCorp, College Station, TX, USA).

## 3. Results

### 3.1. Patient Characteristics

A total of 538 patients met the inclusion criteria, of whom 305 were diagnosed with CTS and 233 served as controls. There was no age distribution difference between patients with and without CTS. There were more males among the patients without CTS than among those with CTS. Patients without CTS presented to the CNPL with shorter disease durations than patients with CTS. DM was present in similar proportions among patients with and without CTS. A third of the patients without CTS had mainly ulnar neuropathy and cervical radiculopathy, whereas the rest exhibited other musculoskeletal etiologies. Table 2 summarizes the patients’ baseline demographic and clinical characteristics.

### 3.2. Diagnostic Accuracy for Patients Without DM

Regarding the single COLSs, palmdiff had the highest sensitivity, specificity, PPV, and NPV. The single abnormal COLS produced PPVs ranging from 87.6% to 92.6%. Two abnormal COLSs consistently produced PPVs of 94.1–94.8% and a specificity range of 95.7–97.3%. The results were similar across different combinations of COLSs. Two normal COLSs consistently produced NPVs of 61.7–64.5%, with a sensitivity range of 57.8–61.9%. Table 3 lists details on diagnostic accuracy; the numbers in bold represent the PPV/specificity range of two abnormal COLSs and the NPV/sensitivity of two normal COLSs. The kappa statistic demonstrates substantial agreement between comparative latency studies (COLSs) as well as between single and two concordant COLSs. Despite this agreement, their performances were not equivalent, as McNemar’s test indicates a significant difference in the diagnostic classification of cases between single and two concordant COLSs (see Supplementary Tables S1 and S2).

### 3.3. Diagnostic Accuracy for Patients with DM

Regarding single COLS, ringdiff had the highest specificity and PPV, whereas thumbdiff had the highest sensitivity and NPV. Two abnormal COLSs produced PPVs of 88.5–91.7% and a specificity of 93.8–95.8%. Two normal COLSs produced a NPV of 61.1–65.2% and sensitivity of 54.1% to 62.3% (Supplementary Table S3). Among patients with DM, a burning sensation was reported in 12 (28.57%) and 27 (49.09%) patients without CTS and with CTS, respectively. The kappa statistic demonstrates moderate to substantial agreement between the comparative latency studies (COLSs) as well as between single and two concordant COLSs. However, their performance was different, as McNemar’s test showed a significant difference in the diagnostic classification of cases between single and two concordant COLSs (see Supplementary Tables S1 and S2).

Because PPV did not surpass 92% for patients with DM, we further investigated diagnostic accuracy based on age group. For patients <60 years old, the single COLSs palmdiff had the highest specificity and PPV, whereas thumbdiff had the highest sensitivity and NPV (Table 4). Two abnormal COLSs produced PPVs of 94.1–95.0% and a specificity of 94.7%. Two normal COLSs produced NPV of 56.3–64.0% and a sensitivity of 63.2–76.3%. Table 4 lists the details of diagnostic accuracy; the numbers in bold represent the PPV/specificity range of two abnormal COLSs and the NPV/sensitivity of two normal COLSs. For patients ≥60 years old, neither a single nor two abnormal COLSs reached a PPV of 85% or more. Two abnormal COLSs produced PPVs of 71.4–75% and a specificity of 93.1–96.6%. Two normal COLSs produced NPVs of 63.4–67.5% and a sensitivity 34.8–43.5%. Table 4 lists the details of diagnostic accuracy; the numbers in bold represent the PPV/specificity range of two abnormal COLSs and the NPV/sensitivity range of two normal COLSs.

### 3.4. Diagnostic Accuracy for Patients with Symptom Durations Greater than Six Months

For patients without DM (*n* = 269), two abnormal COLSs consistently produced PPVs of 100%, whereas the NPV of two normal COLSs ranged from 52.1% to 53.6% (Table 3). For patients with DM, the diagnostic accuracy of COLSs did not improve for patients aged <60 years (*n* = 29), and it was reduced for patients aged ≥60 years (*n* = 29) (Supplementary Table S4).

## 4. Discussion

This study evaluated the diagnostic accuracy of a single COLS and the concordance between two COLSs for confirming the diagnosis of CTS. Our study used a non-selective clinic population, and we present the results stratified by the presence or absence of DM, which has not been reported separately in the literature. The role of COLSs in confirming CTS has been emphasized in more than sixteen studies; however, only a few have examined the diagnostic value of using multiple COLSs, and several limitations have been identified in these studies, as outlined in the Introduction [3,21]. Among patients without DM, we observed that requiring concordance between two COLSs consistently confirmed CTS with a PPV of >94%, whereas single COLS had varied capacities for confirming CTS, with PPVs of 87.6–92.6%. Despite substantial agreement between single and two concordant COLSs, McNemar’s test suggests that they do not perform equivalently in classifying patients into CTS and non-CTS cases. When we included only patients with symptom durations of >6 months, two abnormal COLSs consistently achieved a PPV of 100% using our predetermined cutoff values. This is consistent with previous reports that recommend agreement between two comparison tests to confirm a CTS diagnosis [1,22]. Robinson et al. observed a PPV for two COLSs of 98%, and an NPV of 79%, which is higher than the predictive value of a single COLS [23]. They also found that the PPV of the combined sensory index (CSI)—which is the sum of palmdiff, thumbdiff, and ringdiff—was 98%, which is higher than our reported PPV for two abnormal COLSs. We previously reported a PPV for the CSI of 90% among patients without DM [20]. The reported pooled specificity for single COLS was 79–99%, whereas our study observed a specificity of 93–94% for single COLS and 95–97% for two concordant COLSs [11]. A possible explanation for the difference in predictive values is that Robinson et al. used a defined number of asymptomatic controls rather than including non-selected clinic populations. This may not reflect the real prevalence of CTS and may thus subsequently weaken the accuracy of predictive values, which are largely influenced by disease prevalence [12]. In contrast, by including a non-selective clinic population—presumably encompassing groups such as manual laborers and those with high body weights in proportions that reflect their prevalence in the general population—our study design likely reflects the real prevalence of the disease and accounts for the fact that asymptomatic median nerve compression at the wrist is relatively common, occurring in up to 20% of individuals [24]. Considering our data and the available literature, we propose that the use of two concordant COLSs modifies the diagnostic categorization in some cases, potentially improving the accuracy of carpal tunnel syndrome confirmation among patients without DM.

Among patients with DM younger than 60 years old, we observed that requiring concordance between two COLSs consistently confirmed CTS with a PPV of >94%, whereas a single COLS exhibited a varied capacity to confirm CTS, with a PPV range of 89.3–95.8%. No prior studies have reported the PPV and NPV of CTS electrodiagnostic studies for patients with DM. However, the CSI has been reported to vary from 91% to 100% for patients with DM under the age of 60 years [20]. For patients with DM who are 60 years or older, the PPV dropped significantly to 71–83% for a single COLS or concordance between two COLSs; however, the NPV remained at 58–67%. These values were similar to the CSI for patients with DM who were over the age of 60 years [20]. By reporting diagnostic accuracy for patients with DM, our study addresses an important aspect that has previously been neglected, since the available literature suggests that it is very important to consider different cutoff values and diagnostic accuracy assessments for patients with DM compared to patients without DM. In a large multi-center trial, 23% of patients demonstrated median neuropathy at the wrist if conventional EDX parameters were applied [1,25]. The authors suggest that different EDX parameters should be used to diagnose CTS in patients with DM, as latencies could be prolonged even in the absence of CTS [25]. A prior study showed that ringdiff had a high specificity and sensitivity for CTS diagnosis among patients with DM, but the authors did not consider the effect of age and did not report the palmdiff or thumbdiff studies’ specificity and sensitivity [6]. Another study found that ringdiff had a high sensitivity of 90% and a moderate specificity of 85%, whereas thumbdiff had a sensitivity of 82% and a specificity of 80% among patients with DM [26]. This high sensitivity is largely attributable to the low cutoff value of 0.35 ms that was applied, which, however, comes at the expense of reduced specificity. These studies did not report PPV and NPV results, probably due to the nature of control group selection. Taking all of the data together, we find that concordance between two COLSs provides more accurate confirmation of CTS among patients with DM under 60 years old.

There are four key reasons why it is particularly important to evaluate the diagnostic accuracy of CTS testing in patients with DM while simultaneously accounting for the effect of aging. First, DM has been identified as an independent risk factor for CTS even when using a method that is less likely to be influenced by confounders, such as Mendelian randomization analysis [27]. Second, aging affects the median nerve at the wrist more than other nerves outside the entrapment site [20,28,29]. Third, patients with DM and CTS have weaker hand grip strength and poorer dexterity when compared to patients with DM and without CTS [30]. Fourth, several studies have shown that DM does not mitigate the benefits of CTS decompression surgery, and surgery may prevent weakness [31,32]. These considerations highlight the need to better understand the role of EDX tests in confirming CTS diagnosis among patients with diabetes. This is particularly important because EDX testing may offer greater accuracy than ultrasound use in this population [6]. Such a better understanding will lead to more accurate diagnoses and prevent functional limitations among patients with DM and CTS. When only patients with symptom durations of >6 months were included, diagnostic accuracy did not improve in patients with DM aged <60 years, and it was reduced in those with DM aged ≥60 years. The likely explanation for this is the smaller sample sizes of patients in these two groups. The reason for these smaller sample sizes is probably related to absent sensory responses in EDX tests with longer symptom duration, particularly in patients with DM over the age of 60 years. In such a group, the use of the lumbrical–interosseous median–ulnar distal latency difference has been shown to improve diagnostic accuracy [33].

From a practical perspective, several factors must be considered when choosing between requiring a single COLS or two concordant COLSs. Our study shows that two concordant tests consistently confirm CTS diagnosis with a PPV of >94% among patients without DM and among patients with DM under 60 years old. In contrast, some single COLSs could have a high PPV, but that was not consistent across all single COLS. Although the improvement in PPV with two concordant COLSs over a single COLS is modest, when nerve conduction studies are used to confirm CTS prior to surgery—as recommended by some guidelines—the use of concordant COLSs may be more appropriate for the following reasons [24,34]. First, single diagnostic tests are vulnerable to random technical errors and chance findings, limiting their reliability, whereas concordance among multiple EDX measures enhances consistency by canceling out nonsystematic errors. This approach also reduces false positives, as it is less likely that several independent parameters in a healthy subject would all show inaccurate values in the same direction. Second, the need for more accurate diagnosis is also emphasized, as one study found that 10–15% of patients who underwent carpal tunnel release surgery were unsatisfied with the outcomes, likely due to inaccurate diagnose [35].

Several limitations should be acknowledged. First, although we applied standardized clinical criteria for CTS classification, which is accepted in research practice, misclassification remains a possibility. Second, the study did not explore the effect of other possible factors that may influence the EDX findings, such as occupation and body weight. Third, our study did not include longitudinal follow-up data that would permit investigating the value of EDX findings in predicting responses to CTS therapeutic options. Fourth, our study did not include other EDX tests for CTS, such as the first lumbrical to second interosseous median to ulnar motor latency difference. Fifth, among patients with DM, we did not investigate the role of the presence of neuropathy and its influence on the EDX findings. Sixth, our study addresses patients with mild-to-moderate CTS who may represent diagnostic challenges while we excluded cases of severe CTS where sensory responses were absent. Seventh, we did not include symptoms in the median nerve distribution or presence of Phalen’s sign as part of the diagnostic criteria. Finally, the generalizability of our findings may be constrained by the limited number of participating centers.

In conclusion, this study shows that there is substantial agreement between single COLS and two concordant COLSs; however, they did not perform equivalently in the diagnostic classification of cases into CTS and non-CTS. Concordance between two COLSs increases specificity and PPV in diagnosing CTS, particularly among patients without DM and patients with DM under 60 years old. These results may support incorporating two concordant COLSs into diagnostic protocols, especially for surgical decision-making. For patients with DM who are ≥60 years of age, diagnostic accuracy did not improve with the use of two abnormal COLSs, and using the lumbrical–interosseous median–ulnar distal latency difference may offer an alternative, as proposed in previous studies [33]. Future studies could investigate the value of EDX findings in predicting the response to CTS therapeutic options.

## Figures and Tables

**Table 1:** Comparative latency study cutoff values.

	Without DM	With DM
Palmdiff	≥0.5 ms	≥0.5 ms (<50 years) ≥1.2 ms (≥50 years)
Ringdiff	≥0.6 ms	≥0.5 ms (<50 years) ≥1.6 ms (≥50 years)
Thumbdiff	≥1 ms	≥1 ms (<60 years) ≥1.8 ms (≥60 years)

**Table 2:** Patient characteristics.

Variable	Patients Without CTS n = 233	Patients with CTS n = 305
Age (years), median (IQR)	45 (35–57)	49 (40–56)
Male, n (%)	113 (48.50%)	53 (17.38%)
Diabetes Mellitus, n (%)	48 (20.60%)	61 (20.00%)
Hypothyroidism, n (%)	19 (9.55%)	57 (19.39%)
Disease duration (months), median (IQR)	6 (2–24)	12 (5–36)
Burning feet	48 (22.64%)	106 (37.46%)
Right-hand dominant, n (%)	144 (92.31%)	190 (92.23%)
Age < 30 years, n (%)	35 (15.02%)	22 (7.21%)
Age 30–39 years, n (%)	46 (19.74%)	54 (17.70%)
Age 40–49 years, n (%)	52 (22.32%)	77 (25.25%)
Age 50–59 years, n (%)	50 (21.46%)	104 (34.10%)
Age≥ >60 years, n (%)	50 (21.46%)	48 (15.74%)
Padua CTS severity classification		
Padua 0, n (%)	71 (30.47%)	35 (11.48%)
Padua 1, n (%)	28 (12.02%)	25 (8.20%)
Padua 2, n (%)	112 (48.07%)	114 (37.38%)
Padua 3, n (%)	22 (9.44%)	131 (42.95%)
Bland CTS severity classification		
Bland 0, n (%)	147 (63.09%)	100 (32.79%)
Bland 1, n (%)	36 (15.45%)	44 (14.43%)
Bland 2, n (%)	46 (19.74%)	96 (31.48%)
Bland 3, n (%)	4 (1.72%)	65 (21.31%)
Distribution of CTS and non-CTS diagnoses		
CTS, n (%)	0 (0.00%)	305 (100.00%)
Ulnar neuropathy, n (%)	43 (18.45%)	0 (0.00%)
Radiculopathy, n (%)	34 (14.59%)	0 (0.00%)
Rotator cuff, n (%)	6 (2.58%)	0 (0.00%)
De Quervain’s tenosynovitis, n (%)	5 (2.15%)	0 (0.00%)
Carpometacarpal arthritis, n (%)	6 (2.58%)	0 (0.00%)
Fibromyalgia, n (%)	12 (5.15%)	0 (0.00%)
Epicondylitis, n (%)	7 (3.00%)	0 (0.00%)
Musculoskeletal disorders, n (%)	120 (51.5%)	0 (0.00%)

IQR: interquartile range. CTS: carpal tunnel syndrome.

**Table 3:** Diagnostic accuracy of single and concordant COLSs among patients without DM.

COLS	ROC (95% CI)	Sensitivity (95% CI)	Specificity (95% CI)	PPV (95% CI)	NPV (95% CI)
	Whole cohort (n = 429)				
Thumbdiff	0.683 (0.65–0.72)	43% (36.7–49.5)	93.5% (88.9–96.6)	89.7% (82.8–94.6)	55.4% (49.7–61)
Palmdiff	0.753 (0.72–0.79)	56.6% (50.1–62.9)	94.1% (89.6–97)	92.6% (87.2–96.3)	62.1% (56.2–67.8)
Ringdiff	0.73 (0.69–0.77)	52.5% (46–58.9)	93.5% (88.9–96.6)	91.4% (85.5–95.5)	59.9% (54–65.6)
One of three COLSs	0.758 (0.72–0.79)	63.5% (57.1–69.6)	88.1% (82.6–92.4)	87.6% (81.8–92)	64.7% (58.4–70.6)
Two of three COLSs	0.741 (0.71–0.78)	52.5% (46–58.9)	95.7% (91.7–98.1)	**94.1% (88.7–97.4)**	60.4% (54.6–66)
One of palmdiff–thumbdiff	0.753 (0.72–0.79)	59.8% (53.4–66)	90.8% (85.7–94.6)	89.6% (83.8–93.8)	63.2% (57.1–69)
Two of palmdiff–thumbdiff	0.683 (0.65–0.72)	39.8% (33.6–46.2)	96.8% (93.1–98.8)	94.2% (87.8–97.8)	54.9% (49.3–60.4)
One of palmdiff–ringdiff	0.766 (0.73–0.80)	61.9% (55.5–68)	91.4% (86.3–95)	90.4% (84.9–94.4)	**64.5% (58.4–70.3)**
Two of palmdiff–ringdiff	0.717 (0.68–0.75)	47.1% (40.7–53.6)	96.2% (92.4–98.5)	94.3% (88.5–97.7)	58% (52.2–63.6)
One of thumbdiff–ringdiff	0.738 (0.70–0.78)	57.8% (51.3–64.1)	89.7% (84.4–93.7)	88.1% (82.1–92.7)	**61.7% (55.6–67.5)**
Two of thumbdiff–ringdiff	0.675 (0.64–0.71)	37.7% (31.6–44.1)	97.3% (93.8–99.1)	**94.8% (88.4–98.3)**	54.2% (48.7–59.7)
	Patients with symptom duration > 6 months (n = 269)				
Thumbdiff	0.669 (0.63–0.71)	37.6% (30.2–45.4)	96.2% (90.4–98.9)	93.9% (85.2–98.3)	49.3% (42.2–56.4)
Palmdiff	0.668 (0.63–0.71)	34.5% (27.3–42.3)	99.0% (94.8–100)	98.3% (90.8–100)	48.8% (41.9–55.8)
Ringdiff	0.684 (0.64–0.73)	40.6% (33.0–48.5)	96.2% (90.4–98.9)	94.4% (86.2–98.4)	50.5% (43.3–57.7)
One of three COLSs	0.717 (0.67–0.76)	52.1% (44.2–59.9)	91.3% (84.2–96.0)	90.5% (82.8–95.6)	54.6% (46.9–62.1)
Two of three COLSs	0.685 (0.65–0.72)	37.0% (29.6–44.8)	100% (96.5–100)	**100% (94.1–100)**	50.0% (43.0–57.0)
One of palmdiff–thumbdiff	0.709 (0.67–0.75)	46.7% (38.9–54.6)	95.2% (89.1–98.4)	93.9% (86.3–98.0)	52.9% (45.5–60.3)
Two of palmdiff–thumbdiff	0.627 (0.59–0.66)	25.5% (19.0–32.8)	100% (96.5–100)	**100% (91.6–100)**	45.8% (39.2–52.5)
One of palmdiff–ringdiff	0.700 (0.66–0.74)	44.8% (37.1–52.8)	95.2% (89.1–98.4)	93.7% (85.8–97.9)	**52.1% (44.8–59.4)**
Two of palmdiff–ringdiff	0.652 (0.62–0.69)	30.3% (23.4–37.9)	100% (96.5–100)	**100% (92.9–100)**	47.5% (40.7–54.3)
One of thumbdiff–ringdiff	0.710 (0.66–0.76)	49.7% (41.8–57.6)	92.3% (85.4–96.6)	91.1% (83.2–96.1)	**53.6% (46.0–61.1)**
Two of thumbdiff–ringdiff	0.642 (0.61–0.68)	28.5% (21.7–36.0)	100% (96.5–100)	**100% (92.5–100)**	46.8% (40.1–53.6)

PPV: positive predictive value. NPV: negative predictive value. COLSs: comparative latency studies. Palmdiff: the median–ulnar mixed palmar latency difference test. Thumbdiff: the median–radial thumb latency difference test. Ringdiff: the median–ulnar ring finger latency difference test. The numbers in bold represent the PPV/specificity range of two abnormal COLSs and the NPV/sensitivity range of two normal COLSs.

**Table 4:** Diagnostic accuracy of single and concordant COLSs among patients with DM.

COLS	ROC (95% CI)	Sensitivity (95% CI)	Specificity (95% CI)	PPV (95% CI)	NPV (95% CI)
**Age < 60 years (n = 57)**					
Thumbdiff	0.750 (0.64–0.86)	65.8% (48.6–80.4)	84.2% (60.4–96.6)	89.3% (71.8–97.7)	55.2% (35.7–73.6)
Palmdiff	0.776 (0.68–0.87)	60.5% (43.4–76.0)	94.7% (74.0–99.9)	95.8% (78.9–99.9)	54.5% (36.4–71.9)
Ringdiff	0.724 (0.63–0.82)	50.0% (33.4–66.6)	94.7% (74.0–99.9)	95.0% (75.1–99.9)	48.6% (31.9–65.6)
One of three COLSs	0.816 (0.71–0.92)	78.9% (62.7–90.4)	84.2% (60.4–96.6)	90.9% (75.7–98.1)	66.7% (44.8–84.4)
Two of three COLSs	0.750 (0.66–0.85)	55.3% (38.3–71.4)	94.7% (74.0–99.9)	95.5% (77.2–99.9)	51.4% (34.0–68.6)
One of palmdiff–thumbdiff	0.803 (0.69–0.91)	76.3% (59.8–88.6)	84.2% (60.4–96.6)	90.6% (75.8–98.0)	**64.0% (42.5–82.0)**
Two of palmdiff–thumbdiff	0.724 (0.63–0.82)	50.0% (33.4–66.6)	94.7% (74.0–99.9)	**95.0% (75.1–99.9)**	48.6% (31.9–65.6)
One of palmdiff–ringdiff	0.789 (0.69–0.88)	63.2% (46.0–78.2)	94.7% (74.0–99.9)	96.0% (79.6–99.8)	**56.3% (37.7–73.6)**
Two of palmdiff–ringdiff	0.711 (0.62–0.81)	47.4% (31.0–64.2)	94.7% (74.0–99.9)	94.7% (74.0–99.9)	47.4% (31.0–64.2)
One of thumbdiff–ringdiff	0.789 (0.68–0.90)	73.7% (56.9–86.6)	84.2% (60.4–96.6)	90.3% (74.2–98.0)	61.5% (40.6–79.8)
Two of thumbdiff–ringdiff	0.684 (0.59–0.78)	42.1% (26.3–59.2)	94.7% (74.0–99.9)	**94.1% (71.3–99.9)**	45.8% (29.3–61.5)
**Age ≥ 60 years (n = 52)**					
Thumbdiff	0.591 (0.49–0.68)	21.7% (7.46–43.7)	96.6% (82.2–99.9)	83.3% (35.9–99.6)	60.9% (45.4–74.9)
Palmdiff	0.579 (0.47–0.69)	26.1% (10.2–48.4)	89.7% (72.6–97.8)	66.7% (29.9–92.5)	60.6% (44.4–75.8)
Ringdiff	0.639 (0.53–0.75)	34.8% (16.4–57.3)	93.1% (77.2–99.2)	80.0% (44.8–97.5)	64.4% (48.8–78.5)
One of three COLSs	0.666 (0.55–0.78)	43.5% (23.2–65.5)	89.7% (72.6–97.8)	76.9% (46.2–95.0)	66.7% (49.8–80.9)
Two of three COLSs	0.618 (0.51–0.73)	30.4% (13.2–52.9)	93.1% (77.2–99.2)	77.8% (40.8–97.2)	62.9% (46.7–77.8)
One of palmdiff–thumbdiff	0.622 (0.51–0.74)	34.8% (16.4–57.3)	89.7% (72.6–97.8)	72.7% (39.0–94.0)	**63.4% (46.9–77.9)**
Two of palmdiff–thumbdiff	0.548 (0.47–0.63)	13% (2.78–33.6)	96.6% (82.2–99.9)	**75% (19.4–99.4)**	58.3% (43.2–72.5)
One of palmdiff–ringdiff	0.644 (0.53–0.76)	39.1% (19.7–61.6)	89.7% (72.6–97.8)	75% (42.8–94.5)	65% (48.3–79.4)
Two of palmdiff–ringdiff	0.574 (0.48–0.67)	21.7% (7.46–43.7)	93.1% (77.2–99.2)	**71.4% (29.8–96.3)**	60% (44.4–74.3)
One of thumbdiff–ringdiff	0.683 (0.57–0.79)	43.5% (23.2–65.5)	93.1% (77.2–99.2)	83.3% (51.6–97.9)	**67.5% (50.9–81.4)**
Two of thumbdiff–ringdiff	0.548 (0.47–0.63)	13% (2.78–33.6)	96.6% (82.2–99.9)	75% (19.4–99.4)	58.3% (43.2–72.5)

PPV: positive predictive value. NPV: negative predictive value. COLSs: comparative latency studies. Palmdiff: the median–ulnar mixed palmar latency difference test. Thumbdiff: the median–radial thumb latency difference test. Ringdiff: the median–ulnar ring finger latency difference test. The numbers in bold represent the PPV/specificity range of two abnormal COLSs and the NPV/sensitivity range of two normal COLS.

## Data Availability

All data are available upon direct request to the corresponding author for the accurate use of the data.

## Supplementary Materials

The following supporting information can be downloaded at: https://www.mdpi.com/article/10.3390/diagnostics15222888/s1, Table S1. Agreement between Comparative latency studies (COLS); Table S2. Comparison between single and concordant Comparative latency studies (COLS) using McNemar’s test; Table S3. Diagnostic accuracy of single and concordant COLS among patients with DM (whole group: not divided by age); Table S4. Diagnostic accuracy of single and concordant COLS among patients with DM, Patients with symptoms duration >6 months

## Abbreviations

The following abbreviations are used in this manuscript:

CTS	Carpal tunnel syndrome
EDX	Electrodiagnostic studies
COLS	Comparative latency studies
Palmdiff	Median–ulnar mixed palmar latency difference test
Ringdiff	Median–ulnar ring finger latency difference test
Thumbdiff	Median–radial thumb latency difference test
