# Supplementary material for: Diagnostic Accuracy of Electrodiagnostic Comparative Latency Studies of Carpal Tunnel Syndrome: Single Test and Concordance Between Multiple Tests
Source: Diagnostics (Basel). 2025 Nov 14;15(22):2888. doi: 10.3390/diagnostics15222888 (PMC12650925; doi:10.3390/diagnostics15222888)
Supplement: Supplementary file 1 [file diagnostics-15-02888-s001.zip › diagnostics-3936419-supplementary.pdf]

Supplementary tables:

Table S1 Agreement between Comparative latency studies (COLS)

Test_A	Test_B	Observed_Agreement(%)	Kappa	p_value
**Patients without DM**				
Palmdiff	Thumbdiff	86.01	0.6752	<0.001
Palmdiff	Ringdiff	89.51	0.7653	<0.001
Thumbdiff	RIingdiff	85.31	0.6512	<0.001
One out of three COLS	Two out of three COLS	90.44	0.7958	<0.001
One of palmdiff-thumbdiff	Two of palmdiff-thumbdiff	86.01	0.6804	<0.001
One of palmdiff -ringdiff	Two of palmdiff -ringdiff	89.51	0.7681	<0.001
One of thumbdiffl-ringdiff	Two of thumbdiffl-ringdiff	85.31	0.6588	<0.001
**Patients with DM**				
Palmdiff	Thumbdiff	82.57	0.5906	<0.001
Palmdiff	Ringdiff	79.82	0.5142	<0.001
Thumbdiff	RIingdiff	89.91	0.7547	<0.001
One out of three COLS	Two out of three COLS	86.24	0.7049	<0.001
One of palmdiff-thumbdiff	Two of palmdiff-thumbdiff	82.57	0.6047	<0.001
One of palmdiff -ringdiff	Two of palmdiff -ringdiff	89.91	0.7574	<0.001
One of thumbdiffl-ringdiff	Two of thumbdiffl-ringdiff	79.82	0.5362	<0.001

COLS: comparative latency studies. Palmdiff: the median–ulnar mixed palmar latency difference test. Thumbdiff median–radial thumb latency difference test. Ringdiff: median–ulnar ring finger latency difference test.

Table S2 Comparison between single and concordant Comparative latency studies (COLS) using McNemar's test

Test_A	Test_B	mcnemar_chi2	p_value
**Patients without DM**			
One out of three COLS	Two out of three COLS	41	<0.001
One of palmdiff-thumbdiff	Two of palmdiff-thumbdiff	60	<0.001
One of palmdiff -ringdiff	Two of palmdiff -ringdiff	45	<0.001
One of thumbdiffl-ringdiff	Two of thumbdiffl-ringdiff	63	<0.001
**Patients with DM**			
One out of three COLS	Two out of three COLS	15	<0.001
One of palmdiff-thumbdiff	Two of palmdiff-thumbdiff	19	<0.001
One of palmdiff -ringdiff	Two of palmdiff -ringdiff	11	<0.001
One of thumbdiffl-ringdiff	Two of thumbdiffl-ringdiff	22	<0.001

COLS: comparative latency studies. Palmdiff: the median–ulnar mixed palmar latency difference test. Thumbdiff median–radial thumb latency difference test. Ringdiff: median–ulnar ring finger latency difference test.

Table S3 Diagnostic accuracy of single and concordant COLS among patients with DM (whole group: not divided by age)

Group/Test	ROC (95% CI)	Sensitivity (95% CI)	Specificity (95% CI)	PPV (95% CI)	NPV (95% CI)
Thumbdiff	0.704 (0.63–0.77)	49.2% (36.1–62.3)	91.7% (80–97.7)	88.2% (72.5–96.7)	58.7% (46.7–69.9)
Palmdiff	0.696 (0.62–0.77)	47.5% (34.6–60.7)	91.7% (80–97.7)	87.9% (71.8–96.6)	57.9% (46–69.1)
Ringdiff	0.69 (0.61–0.76)	44.3% (31.5–57.6)	93.8% (82.8–98.7)	90% (73.5–97.9)	57% (45.3–68.1)
One of three COLS	0.765 (0.68–0.84)	65.6% (52.3–77.3)	87.5% (74.8–95.3)	87% (73.7–95.1)	66.7% (53.7–78)
Two of three COLS	0.698 (0.62–0.77)	45.9% (33.1–59.2)	93.8% (82.8–98.7)	90.3% (74.2–98)	57.7% (46–68.8)
One of palmdiff-thumbdiff	0.741 (0.66–0.81)	60.7% (47.3–72.9)	87.5% (74.8–95.3)	86% (72.1–94.7)	63.6% (50.9–75.1)
Two of palmdiff-thumbdiff	0.659 (0.59–0.72)	36.1% (24.2–49.4)	95.8% (85.7–99.5)	91.7% (73–99)	54.1% (43–65)
One of palmdiff -ringdiff	0.729 (0.65–0.80)	54.1% (40.8–66.9)	91.7% (80–97.7)	89.2% (74.6–97)	61.1% (48.9–72.4)
Two of palmdiff-ringdiff	0.657 (0.58–0.72)	37.7% (25.6–51)	93.8% (82.8–98.7)	88.5% (69.8–97.6)	54.2% (42.9–65.2)
One of thumbdiffl-ringdiff	0.759 (0.68–0.83)	62.3% (49–74.4)	89.6% (77.3–96.5)	88.4% (74.9–96.1)	65.2% (52.4–76.5)
Two of thumbdiff-ringdiff	0.635 (0.57–0.70)	31.1% (19.9–44.3)	95.8% (85.7–99.5)	90.5% (69.6–98.8)	52.3% (41.4–63)

PPV: positive predictive value. NPV: negative predictive value. COLS: comparative latency studies. Palmdiff: the median–ulnar mixed palmar latency difference test. Thumbdiff median–radial thumb latency difference test. Ringdiff: median–ulnar ring finger latency difference test.

Table S4 Diagnostic accuracy of single and concordant COLS among patients with DM, Patients with symptoms duration > 6 months

Test	ROC (95% CI)	Sensitivity (95% CI)	Specificity (95% CI)	PPV (95% CI)	NPV (95% CI)
	**Age <60 years**				
Thumbdiff	0.715 (0.45–0.97)	68% (46.5–85.1)	75% (19.4–99.4)	94.4% (72.7–99.9)	27.3% (6.02–61.0)
Palmdiff	0.715 (0.45–0.97)	68% (46.5–85.1)	75% (19.4–99.4)	94.4% (72.7–99.9)	27.3% (6.02–61.0)
Ringdiff	0.615 (0.35–0.88)	48% (27.8–68.7)	75% (19.4–99.4)	92.3% (64.0–99.8)	18.8% (4.05–45.6)
One of three COLS	0.775 (0.51–1.00)	80% (59.3–93.2)	75% (19.4–99.4)	95.2% (76.2–99.9)	37.5% (8.52–75.5)
Two of three COLS	0.675 (0.41–0.93)	60% (38.7–78.9)	75% (19.4–99.4)	93.8% (69.8–99.8)	23.1% (5.04–53.8)
One of palmdiff-thumbdiff	0.775 (0.51–1.00)	80% (59.3–93.2)	75% (19.4–99.4)	95.2% (76.2–99.9)	37.5% (8.52–75.5)
Two of palmdiff-thumbdiff	0.655 (0.39–0.91)	56% (34.9–75.6)	75% (19.4–99.4)	93.3% (68.1–99.8)	21.4% (4.66–50.8)
One of palmdiff -ringdiff	0.715 (0.45–0.97)	68% (46.5–85.1)	75% (19.4–99.4)	94.4% (72.7–99.9)	27.3% (6.02–61.0)
Two of palmdiff-ringdiff	0.615 (0.35–0.88)	48% (27.8–68.7)	75% (19.4–99.4)	92.3% (64.0–99.8)	18.8% (4.05–45.6)
One of thumbdiffl-ringdiff	0.735 (0.47–0.99)	72% (50.6–87.9)	75% (19.4–99.4)	94.7% (74.0–99.9)	30.0% (6.67–65.2)
Two of thumbdiff-ringdiff	0.595 (0.33–0.85)	44% (24.4–65.1)	75% (19.4–99.4)	91.7% (61.5–99.8)	17.6% (3.80–43.4)
	**Age ≥60 years**				
Thumbdiff	0.584 (0.45–0.71)	23.1% (5.0–53.8)	93.8% (69.8–99.8)	75.0% (19.4–99.4)	60.0% (38.7–78.9)
Palmdiff	0.553 (0.40–0.69)	23.1% (5.0–53.8)	87.5% (61.7–98.4)	60.0% (14.7–94.7)	58.3% (36.6–77.9)
Ringdiff	0.514 (0.38–0.64)	15.4% (1.9–45.4)	87.5% (61.7–98.4)	50.0% (6.8–93.2)	56.0% (34.9–75.6)
One of three COLS	0.591 (0.43–0.74)	30.8% (9.1–61.4)	87.5% (61.7–98.4)	66.7% (22.3–95.7)	60.9% (38.5–80.3)
Two of three COLS	0.553 (0.40–0.69)	23.1% (5.0–53.8)	87.5% (61.7–98.4)	60.0% (14.7–94.7)	58.3% (36.6–77.9)
One of palmdiff-thumbdiff	0.591 (0.43–0.74)	30.8% (9.1–61.4)	87.5% (61.7–98.4)	66.7% (22.3–95.7)	60.9% (38.5–80.3)
Two of palmdiff-thumbdiff	0.546 (0.42–0.66)	15.4% (1.9–45.4)	93.8% (69.8–99.8)	66.7% (9.4–99.2)	57.7% (36.9–76.6)
One of palmdiff -ringdiff	0.553 (0.40–0.69)	23.1% (5.0–53.8)	87.5% (61.7–98.4)	60.0% (14.7–94.7)	58.3% (36.6–77.9)
Two of palmdiff-ringdiff	0.514 (0.38–0.64)	15.4% (1.9–45.4)	87.5% (61.7–98.4)	50.0% (6.8–93.2)	56.0% (34.9–75.6)
One of thumbdiffl-ringdiff	0.591 (0.43–0.74)	30.8% (9.1–61.4)	87.5% (61.7–98.4)	66.7% (22.3–95.7)	60.9% (38.5–80.3)
Two of thumbdiff-ringdiff	0.507 (0.41–0.60)	7.7% (0.2–36.0)	93.8% (69.8–99.8)	50.0% (1.3–98.7)	55.6% (35.3–74.5)

PPV: positive predictive value. NPV: negative predictive value. COLS: comparative latency studies. Palmdiff: the median–ulnar mixed palmar latency difference test. Thumbdiff median–radial thumb latency difference test. Ringdiff: median–ulnar ring finger latency difference test.
